# Shared Neuropathological Characteristics of Obesity, Type 2 Diabetes and Alzheimer’s Disease: Impacts on Cognitive Decline

**DOI:** 10.3390/nu7095341

**Published:** 2015-09-01

**Authors:** Jennifer M. Walker, Fiona E. Harrison

**Affiliations:** Division of Diabetes, Endocrinology & Metabolism, Department of Medicine, Vanderbilt University, 2213 Garland Ave., Nashville, TN 37232, USA

**Keywords:** Alzheimer’s disease, type 2 diabetes mellitus, high fat diets, obesity, insulin resistance, inflammation, diet reversal, animal models, humans, cognition

## Abstract

In the past few decades, the prevalence of obesity and type 2 diabetes mellitus (T2DM), as well as older individuals at risk for Alzheimer’s disease (AD), has increased. While the consumption of diets high in fat (total and saturated) have been linked to increased risk of AD, diets rich in antioxidants, polyunsaturated fats, and omega-3 fatty acids are associated with decreased risk. Additionally, AD patients are at increased risk for developing T2DM. Recent research suggests that there are stronger similarities between AD and T2DM than have previously been considered. Here we review the neurocognitive and inflammatory effects of high-fat diet consumption, its relationship to AD, and the treatment potential of dietary interventions that may decrease risk of cognitive decline and other associated neuropathological changes, such as insulin resistance, oxidative stress, and chronic inflammatory processes.

## 1. Introduction

In the past few decades in America, the prevalence of obesity, and in turn, type 2 diabetes mellitus (T2DM; all abbreviations given in Table 1) has increased; and at the same time, an aging population of older individuals at risk for Alzheimer’s disease (AD) has also increased [1,2]. Recent research suggests that there are strong relationships between AD and T2DM [3]. Outside of age and genetic predisposition, obesity is the strongest risk factor for developing insulin resistance and subsequent T2DM. T2DM is characterized by decreased production and/or availability of insulin, insulin resistance (IR), and hyperglycemia (high blood sugar). There is also evidence of chronic peripheral inflammation and increased production of pro-inflammatory cytokines [4,5], oxidative stress [6,7], and cognitive deficits [8,9].

**Table 1:** List of abbreviations (in alphabetical order).

Abbreviations		
**AD**—Alzheimer’s disease	**HFD**—High fat diet	**LRP-1**—Low-density lipoprotein receptor-related protein 1
**AGEs**—Advanced glycation end-products	**IDE**—Insulin degrading enzyme	**MCP-1**—Monocyte chemotactic protein 1
**APP**—Amyloid precursor protein	**IGF-1**—Insulin growth factor 1	**MRI**—Magnetic resonance imaging
**BBB**—Blood-brain barrier	**IHC**—Immunohistochemistry	**MWM**—Morris water-maze
**BDNF**—Brain-derived neurotrophic factor	IKK—I κ-B kinase	**NMDA**—*N*-Methyl-D-aspartate
**CNS** – Central nervous system	**IL**—Interleukin	**PI3K**—Phosphoinositide 3 kinase
**CSF**—Cerebrospinal fluid	**INF-γ**—Interferon **γ**	**PKR**—Protein kinase RNA-activated
**EE**—Environmental enrichment	**i.p.** —intraperitoneal	**PS1**—Presenilin 1
**EGCG**—(-)-epigallocatechin-3-gallate	**IR**—Insulin resistance	**RAGE**—Receptor for advanced glycation end-products
**GFAP**—Glial fibrillary acidic protein	**IRS**—Insulin receptor substrate	**T2DM**—Type 2 diabetes mellitus
**GLP-1**—Glucagon-like peptide 1	**IV**—Intravenous	**TNF**—Tumor necrosis factor
**GLUT4**—Glucose transporter 4	**JNK**—c-Jun NH_2_-terminal kinase	**WB**—Western blotting
**GTT**—Glucose tolerance test	**LFD**—Low fat diet	

**Figure 1:**
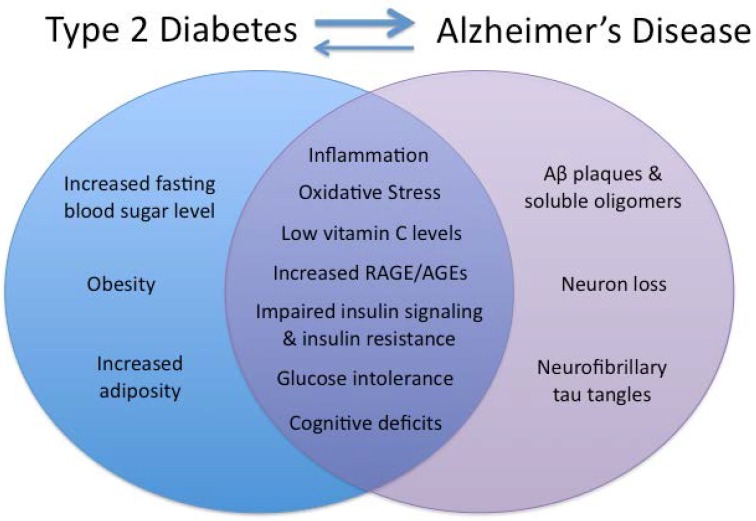
Shared and distinct symptoms of Type 2 Diabetes and Alzheimer’s disease (AD). Venn diagram illustrating the shared and distinct characteristics of type 2 diabetes mellitus (T2DM) and Alzheimer’s disease. In the past few decades, the prevalence of both has increased substantially, and emerging research suggests that there may be a bidirectional relationship between these disease processes.

Obesity, T2DM, and chronic intake of diets high in fat, especially saturated fat, have been linked to reduced cognitive function in a variety of tasks in both older adults [8,9,10,11,12,13] and murine models [14], and all are risk factors for developing dementia [15,16], including AD [3,17,18,19]. Additionally, AD patients are at increased risk for developing T2DM [20]. Emerging research suggests a bidirectional relationship between the two disease states with AD-implicated brain dysfunction in the pathogenesis of T2DM. AD is also associated with increased oxidative stress [21,22,23], chronic inflammation [24,25,26,27], and cognitive deficits [28,29], as well as metabolic disturbances, such as impaired neuronal insulin signaling, impaired cerebral energy metabolism [30] and reduced glucose metabolism [31]. Elucidating these shared characteristics (Figure 1) is a first step towards discovering novel AD treatment targets. Now, research needs to establish whether these shared characteristics, including inflammation, insulin resistance, and cognitive impairments, are fixed or reversible, and test the efficacy of new treatments targeted towards improving those that are found to be reversible. Lifestyle choices such as dietary habits and physical activity represent potentially modifiable AD risk factors. Critically, if some degree of the cognitive impairment seen in AD is due to diet-modifiable factors, as opposed to amyloid-beta (Aβ) and tau pathology and cell death, then interventions could realistically improve cognition and quality of life in a way that is not currently possible with traditional AD treatments.

While the consumption of diets high in total and saturated fats have been linked to increased risk of AD, diets rich in polyunsaturated and omega-3 fatty acids are associated with decreased risk [32,33]. Additionally, diets rich in certain antioxidants may offer protective effects and decrease a person’s risk of developing AD and vascular dementia, among others [16,34,35]. Research involving animal models of AD has found promising, beneficial effects of dietary supplementation with a variety of compounds that have anti-amyloidogenic, antioxidative and/or anti-inflammatory properties, including aged garlic extract [36,37], curcumin [38], (-)-epigallocatechin-3-gallate (EGCG: [39]), Vitamins C and E [40], and ketone body precursors (ketone ester-based diet*:* [41])*.* Additionally, caloric restriction has shown promise by improving spatial learning and memory in the Morris water maze (MWM) in male C57/BL6J mice via regulation of insulin signaling [42], and significantly reducing Aβ deposition in aged [43,44] and relatively younger, transgenic AD mouse models [45,46].

AD is the most common form of dementia in older adults in the US, with vascular dementia (VaD) being the second most common [47]. Classic hallmark characteristics of AD pathology include Aβ plaques and oligomers, and tau neurofibrillary tangles. Aβ is created by the sequential cleavage of amyloid precursor protein (APP) at the N- and C-terminus. VaD is diagnosed clinically by the presence of cerebrovascular disease (*i.e.*, embolisms, aneurysms), in which the blood vessels that supply the brain are damaged, and subsequent cognitive impairment. No distinct diagnostic biomarkers have yet been found, and the clinical diagnostic criteria are still being debated in the field [48]. Obesity and T2DM are both risk factors for developing VaD [49], presumably through their ability to promote cerebrovascular disease, including via their effects on cholesterol and hypertension. Vascular risk factors are associated with both AD and VaD, however, their association with VaD is much stronger [49]. White matter lesion load is also more strongly associated with non-AD dementias, including VaD, in depressed, older adults [50]. While VaD and AD share some characteristics and a potential link with obesity and T2DM, this review is focused on Alzheimer’s disease since its prevalence is higher and diagnostic criteria less controversial than that of vascular dementia.

The specific goals of this review are to discuss the shared characteristics and pathology of AD and T2DM, explain some of the possible pathological mechanisms that can contribute to cognitive dysfunction in both diseases (*i.e.*, insulin resistance and impaired signaling, inflammation), and in light of these points, discuss potential novel treatment targets and interventions that could prove useful in improving cognition and quality of life for those suffering from AD.

## 2. Insulin and Insulin Resistance

**Figure 2:**
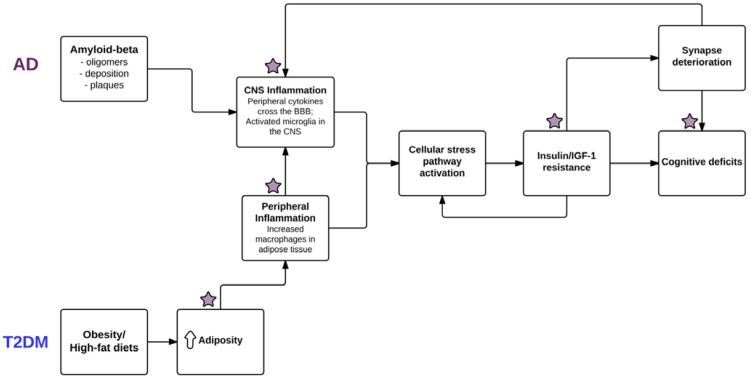
Shared pathological mechanisms of Alzheimer’s disease (AD) and type 2 diabetes mellitus (T2DM). Among the shared pathological mechanisms between the two disease states are inflammatory processes including the release of pro-inflammatory cytokines (some of which can cross the blood-brain barrier (BBB)), e.g., tumor necrosis factor alpha (TNF-α): from microglia in the central nervous system (CNS) in AD, and from macrophages in the periphery in T2DM. In turn, these processes activate cellular stress pathways, including stress kinases I κ-B kinase (IKK) and protein kinase RNA-activated (PKR), which eventually produce insulin/insulin growth factor 1 (IGF-1) resistance via inhibition of insulin receptor substrate 1 (IRS-1) in the CNS (AD) and the periphery (T2DM). Star symbols indicate areas in which diet interventions may exert beneficial effects.

Insulin is a peptide hormone composed of 51 amino acids, produced by β cells in the pancreas. It is critical for glucose homeostasis and metabolism in both the periphery and central nervous system (CNS), by promoting cellular and glial uptake of glucose from the blood. Insulin binds to both its own receptors and insulin-like growth factor-1 receptors (IGF-1R). Insulin receptors are found in high concentrations in the hypothalamus, where they play a role in the regulation of body weight and feeding behavior [51]. Insulin receptors are also located elsewhere in the brain, especially in areas that are important for learning and memory and implicated in AD pathogenesis, such as the cerebral cortex, entorhinal cortex and hippocampus [52]. Insulin-like growth factor-1 (IGF-1) is an endocrine hormone produced mainly in the liver; in the CNS, it acts as a neurotrophic peptide and can promote synaptic plasticity through insulin receptor substrate-1 (IRS-1) activation of the phosphoinositide 3 kinase/protein kinase B (PI3K/Akt) signaling pathway [53,54]. Insulin can also activate this pathway by inducing *tyrosine phosphorylation* of IRS-1. One of the main features of insulin resistance is IRS-1 *serine phosphorylation* [55]. Specifically, insulin signaling is blocked by the activation of c-Jun NH2-terminal kinase (JNK) pathway by tumor necrosis factor-α (TNF-α), which then causes the serine phosphorylation of IRS-1 by various stress-sensitive kinases [56,57]. Serine phosphorylation of IRS-1 then inhibits the tyrosine phosphorylation of IRS-1 and its subsequent binding of PI3K, which is normally induced by insulin stimulation [58], and thereby effectively disrupts insulin signaling within the cell (see Figure 2). Insulin receptor substrate-2 (IRS-2) may also be involved in learning and memory processes. Total IRS-2 deficiency impaired *N*-Methyl-d-aspartate (NMDA) receptor-dependent long-term potentiation at the postsynaptic level in the hippocampus of IRS-2 knockout mice [59]. TNF-α, a pro-inflammatory cytokine, is a common component of inflammatory signaling in AD [60] and T2DM and obesity [61]. In the periphery, TNF-α can be secreted by adipocytes and macrophages; in the brain, TNF-α is mainly secreted by microglial cells. Increased levels of TNF-α are found in both disease processes, and lead to related consequences (*i.e.*, defective insulin signaling in either the peripheral or central nervous systems as a result of the activation of cell stress pathways). Inflammation itself may worsen IR; activation of TNF receptors can cause inhibition of insulin receptor signaling [62].

IR is common in older adults [63]. One consequence of IR and chronic peripheral hyperinsulinemia is down-regulation of insulin transport into the brain, eventually leading to a brain insulin deficient state [64]. In normal older adults, impaired insulin sensitivity has been associated with decreased verbal fluency and decreased grey matter volume in the temporal lobes [65]. Additionally, subtle declarative memory impairments have been observed in middle-aged and older adults with IR prior to the onset of T2DM [66]. IR induces elevations in free fatty acids and inflammatory cytokines in the periphery, which is also exacerbated by obesity [4,67]. Acute hyperinsulinemia (induced by intravenous (IV) insulin infusion) increases Aβ_1-42_ peptide levels in human cerebrospinal fluid (CSF) in an age-dependent fashion in normal, older adults [68], and has pro-inflammatory effects in the CNS (most notably in the hippocampus and hypothalamus) [69]. Moderate hyperinsulinemia (with euglycemia) induced by IV insulin infusion in healthy, older adults increased Aβ_1-42_ levels in blood plasma and CSF, as well as increasing CSF levels of certain pro-inflammatory cytokines: interleukins 1a, 1b, and 6 (IL-1a, IL-1b, IL-6,) and TNF-α [70]. Furthermore, these increases in plasma Aβ_1-42_ were positively correlated with body mass index (BMI), and with levels of TNF-α during the insulin infusion.

Aβ itself can compromise insulin signaling both in the periphery (hepatocytes: [71]) and in the CNS [72] and has been shown to do so using various *in vitro* [71], *in vivo* [72] and *ex vivo* [73] methods and animal models. In cultured hippocampal neurons, Aβ oligomers inhibit insulin signaling [74]. An *in vivo* study using rats [72] showed that intrahippocampal injections of oligomeric Aβ_1-42_ acutely impaired insulin signaling and decreased spontaneous alternation behavior. Twice daily intraperitoneal (i.p.) injections of Aβ_1-42_ in 10-week old, male C57BL/6J mice (on standard lab chow) induced hepatic insulin resistance, reduced hepatic insulin signaling, and increased fasting blood sugar levels [75]. In cynomolgus monkeys, intracerebroventricular injections of oligomeric Aβ led to impaired insulin signaling in the hippocampus [74]. Post-mortem human brains and brains from APP_swe_/PS1_ΔE9_ mice (transgenic model of AD, made by the insertion of human APP and presenilin 1 (PS1) genes known to cause familial AD) also show impaired insulin signaling (measured by immunohistochemistry (IHC) and western blotting (WB) techniques, respectively) in the hippocampus [74]. These effects were found to be mediated by the same mechanisms: activation of the JNK/TNF-α pathway and increased serine phosphorylation of IRS-1/decreased tyrosine phosphorylation of IRS-1. Additionally, the hippocampal formation and the cerebellar cortex of non-diabetic human AD brains show decreased responses to insulin and IGF-1 signaling (*i.e.*, IR/IGF-1 resistance) and elevated basal levels of serine phosphorylated IRS-1, as measured by *ex vivo* stimulation [73]. Basal levels of serine phosphorylated IRS-1 were negatively correlated with measures of working- and episodic memory performance (previously collected from this cohort) even after controlling for Aβ plaques and tau neurofibrillary tangles, which suggests that IR/IGF-1 resistance in the brain may encourage cognitive impairments independently of plaques and tangles.

The drugs currently available on the US market and approved for the treatment of Alzheimer’s disease, such as acetylcholinesterase inhibitors (e.g., donezepil, rivastigmine) and NMDA-receptor antagonists (memantine), cannot reverse or stop AD pathology; at best, they slow the progression of cognitive and behavioral symptoms, but their effectiveness varies across individuals and can decrease over time within the same individual. More recent research in novel treatments for AD has found promising results using medication originally developed to treat diabetes, such as insulin [76,77] and glucagon-like peptide-1 (GLP-1) analogs [74,78,79]. Daily intranasal insulin administration (lasting seven days) reduced levels of Aβ_1-40_ and microglia activation, and improved insulin signaling in the brains in 3xTg mice [80]. A double-blind randomized, placebo-controlled clinical trial for older adults diagnosed with probable mild to moderate AD (*n* = 40) or amnestic mild cognitive impairment (MCI; *n* = 64) found that 4-months of twice daily intranasal insulin administration (20 IU total daily dosage) via a nasal drug delivery device improved delayed memory performance, preserved general cognition (measured by Alzheimer’s Disease’s Assessment Scale-cognitive subscale score) and preserved functional ability (assessed by the ‘activities of daily living scale’ from the Alzheimer’s Disease Cooperative Study) [76]. Continued research is needed to determine how long these beneficial effects may last. Treatment with exendin-4, a GLP-1 analog approved for the treatment of diabetes mellitus, reduces Aβ oligomer-induced JNK activation, IRS-1 serine phosphorylation, and impairment of axonal transport, and improves insulin signaling (by increasing IRS-1 tyrosine phosphorylation) in cultured hippocampal neurons and in the brains of 14-month old, male APP_swe_/PS1_ΔE9_ mice [74]. Mice injected (i.p.) with exendin-4 daily for 3 weeks showed improved acquisition and retention of spatial learning in the MWM, as well as reduced soluble Aβ levels and plaque load in the cerebral cortex.

## 3. Inflammation Is Related to Impaired Insulin Signaling in the Periphery and the CNS

Aβ plaques are associated with the activation of microglia and astrocytes. These cells are involved in normal, beneficial, neuroinflammatory responses; however, their uncontrolled and sustained activation in AD can increase levels of pro-inflammatory cytokines (e.g., TNF-α and interleukins (ILs), such as IL-1b, IL-6), reactive oxygen species and oxidative stress, and lead to secondary neuronal injury and death, which further activate inflammatory processes in a positive feedback loop [81]. Recent research using APP-transgenic mice as an AD mouse model [82] found elevated levels of IL-6 in the CNS in response to systemic peripheral inflammation induced by lipopolysaccharide treatment, as well as increased blood-brain barrier (BBB) permeability for peripheral inflammatory cytokines. Both production and mRNA expression of the cytokines interferon-γ (INF-γ) and interleukin-12 (IL-12) are upregulated in the cortex of Tg2576 mice [83]; additionally, transcription of these same cytokines is increased in the reactive microglia and astrocytes located around Aβ deposits. Aβ oligomers can also impair neuronal insulin signaling [84] via a TNF-α mediated activation of the JNK pathway, and its subsequent inhibition of IRS-1 [85]. RAGE (Receptor for Advanced Glycation End-products) is an immunoglobulin cell surface molecule whose ligands include AGEs (Advanced Glycation End-products) and Aβ fibrils; the presence of these ligands can activate or upregulate the receptor. When Aβ is present, RAGE increases in blood vessels, neurons and microglia, which leads to a highly dysfunctional feedback loop since RAGE transports Aβ across the BBB from the blood into the brain. APP^+^-*ob/ob* mice show an upregulation of RAGE in the brain [86]. In contrast, LRP-1 (low-density lipoprotein receptor-related protein 1) is responsible for the clearance of Aβ from the brain, into the blood. Hyperglycemia also increases the production of AGEs, and AGEs have been identified in Aβ plaques and neurofibrillary tangles through histological staining, indicating a further link between Aβ, RAGE, and oxidative stress response in Alzheimer’s disease [87].

Obesity (in humans: BMI > 30 kg/m^2^) is associated with chronic, low level peripheral inflammation [88]. Obesity and IR can lead to elevated levels of pro-inflammatory cytokines (e.g., TNF-α, IL-6, IL-1b, *etc.*) in both the peripheral and central nervous system of humans (T2DM: [4]) and animals [67,89]. Importantly, certain pro-inflammatory cytokines, like TNF-α and the ILs, can cross the BBB and act on targets in the CNS [90,91]. Adipose (fat) tissue plays a role in pro-inflammatory cytokine secretion; obesity is associated with an increased number of macrophages, which are responsible for most of the TNF-α and IL-6 cytokine production [61]. Furthermore, TNF-α induced activation of JNK results in peripheral IR via its effects on cellular stress kinases (IKK and PKR) and subsequent inhibition of IRS-1 ([85]; see Figure 2).

## 4. High-Fat Diet Feeding, Obesity, and T2DM Can Induce Cognitive Impairments in Non-AD Humans and Animals, and Is Associated with Changes in the Brain

In mice and rats, diet-induced obesity can induce cognitive deficits through its effects on inflammatory and other physiological systems [14,92,93,94]. Impairments of egocentric procedural learning and memory have been found in 12-month old, male C57BL/6J mice maintained on a very high-fat diet (60% kcal fat) using a modified version of the Stone T-maze, in which mice are required to wade (not swim) through a series of corridors with a short ceiling to prevent mice from rearing. This task requires mice to learn a complex sequence of 13 consecutive left/right turns in order to escape to the goal box [92]. Using obese Zucker rats as a model of T2DM, Winocur and colleagues [93] found memory deficits in a hippocampal-dependent, long-interval variable alternation task, as well as impaired hippocampal insulin signaling and decreased expression of GLUT4 (glucose transporter 4). Molteni and colleagues [94] used female Fisher 344 rats to study the effects of a high fat, refined sugar diet (HFS) on brain/neural function and neuronal plasticity in order to evaluate the possible direct effects of this diet on neural function. Female rats are known to *not* develop hypertension or atherosclerosis within the first year of being on a high fat, refined sugar diet, limiting the potential confounds from cardiovascular dysfunction. They found that rats who performed well in the MWM showed increased levels of hippocampal brain-derived neurotrophic factor (BDNF) protein and mRNA. Additionally, they found that just 2 months of a high fat, refined sugar diet was enough to reduce hippocampal BDNF and memory performance in the MWM task.

In humans, obesity has been linked to reduced focal grey matter volume in the frontal lobes [95], as well as impairments in executive function, and learning and memory compared to non-obese individuals [96,97]; cognitive impairments have been demonstrated in obese individuals across almost all domains of cognition that have been studied, including decision making, complex attention, verbal declarative memory, and visual memory, in addition to decreased information processing speed [12]. Obesity in late life is strongly associated with lower verbal abilities [98]. Population-based studies suggest that long periods of stable, consistent obesity across midlife are a better predictor for cognitive impairments across domains later in life compared to obesity measured at a single time point in midlife or late life [98]. This finding suggests that the longer an individual is obese, the more robust the deficits in cognitive ability will be. While it is not yet clear whether obesity itself has a truly independent relationship with these complications, or whether they are entirely caused by obesity-related pathologies (*i.e.*, insulin resistance, hyperglycemia, and chronic inflammation, as seen in T2DM), evidence presented here supports the idea that obesity-induced inflammation and insulin resistance are important factors predicting whether obese individuals will display cognitive impairments or not. A small cross-sectional study using 94 non-demented middle-aged/older adults found that *non-T2DM* individuals with evidence of insulin resistance (*n* = 38) showed decreased executive functioning and deficits in declarative memory compared to matched controls without insulin resistance (*n* = 54) on a neuropsychological test battery [66]. Another large cross-sectional study [65] examined data from 285 cognitively healthy, elderly adults (134 females, 151 males; all 75 years old) originally obtained in the Swedish PIVUS (Prospective Investigation of the Vasculature in Uppsala Seniors) population-based prospective cohort study’s 5-year follow-up [99] and found that insulin resistance (as measured by the homeostasis model assessment of IR method) was negatively correlated with performance on a categorical verbal fluency task, as well as with brain size and grey matter volume in the temporal lobes, as measured by magnetic resonance imaging (MRI).

Observed cognitive impairments are more robust and affect more domains when obese individuals have T2DM and poor glycemic control. However, even T2DM patients with reasonably good glycemic control can show cognitive impairments, as evidenced by a recent, small (88 total participants; 41 with T2DM, 47 matched controls) cross-sectional study which found that non-demented middle-aged T2DM patients with good glycemic control show verbal declarative memory impairments, as well as reduced hippocampal volume, as measured by MRI [100]. Additionally, treated T2DM patients (those currently being treated with medication or dietary restrictions to help improve glucose tolerance) consistently show deficits in verbal memory and information processing speed compared to untreated T2DM individuals with poor glycemic control [9], and certain factors such as microvascular disease, increasing age (especially after 70 years old), early T2DM onset, poor glycemic control, and interactions with dementia can hasten and worsen cognitive deficits. It is important to consider whether the T2DM patients in a given study are being treated for glucose intolerance or insulin resistance since, as mentioned previously, research shows that poor glycemic control can worsen cognitive impairments. A 4-year longitudinal study using MRI measures (106 participants; 68 non-demented T2DM men and women, 38 matched controls) [101] found that T2DM patients who displayed accelerated cognitive decline (25% of T2DM participants) compared to normally aging, matched controls also displayed decreased brain volumes, and increased volumes of the lateral ventricles and white-matter hyperintensities compared to T2DM patients without accelerated cognitive decline across the four year time period; however, no specific vascular or metabolic risk factors for the acceleration were found. Cognitive decline in these participants was most robustly observed in executive function and information processing speed. Structural abnormalities in the brain’s white matter network have also been identified via MRI in non-demented T2DM patients and these abnormalities were also associated with decreased speed in information processing, which was partially independent of vascular brain lesion load [102]. Other brain changes that are associated with T2DM in the context of aging and may contribute to cognitive dysfunction, as found in MRI studies, include greater microvascular disease burden and global brain atrophy, the latter of which is associated with greater amounts of time spent living with T2DM and increased insulin resistance [103].

## 5. Mouse Models of AD Exhibit Greater Sensitivity to Obesity Phenotype and High-Fat Diet

A bigenic mouse model of AD (APP_swe_/PS1_ΔE9_ [104]) was more susceptible to weight gain than wildtype littermates when given a high fat diet (HFD; 55% kcal/fat) [105] for 8 weeks, beginning at 13 months of age. In contrast, body weights of even older APP_swe_/PS1_ΔE9_ mice and wildtype controls (16–17 and 20–21 months old) on a standard, low fat, chow diet, did *not* differ between genotypes. APP_swe_/PS1_ΔE9_ mice also showed fasting hyperglycemia, and lost less weight following a 16-h overnight fast compared to wildtype controls. Both HFD-fed and chow-fed APP_swe_/PS1_ΔE9_ mice demonstrated slower rates of glucose clearance in glucose tolerance tests (GTTs) [105]. By 14-months of age, these mice have developed significant Aβ levels [106,107], as well as exhibiting cognitive and behavioral impairments. Younger (20-week old) male APP_swe_/PS1_ΔE9_ mice on standard chow had increased plasma insulin levels compared to wildtype controls in both fasting (4-h) and fed states. Additionally, mice with higher levels of Aβ_1-40_ and Aβ_1-42_ in plasma showed hyperinsulinemia, impaired insulin signaling in the liver, glucose intolerance, and insulin resistance [71]. Remarkably, male APP_swe_/PS1_ΔE9_ mice began showing reduced glucose clearance in GTTs (at 60 m and 120 m post-injection) as early as 10-weeks old, and by 18-weeks old, they showed higher blood glucose levels (poorer clearance) at all GTT time points (*i.e.*, 15 m, 30 m, 60 m, 120 m post-injection); however, basal blood sugar levels (pre-injection) did not differ between genotypes at either 10- or 18-weeks old.

APP23 mice crossed with the *ob/ob* mouse model for diabetes show earlier learning and memory deficits in the hidden platform version of the MWM at 2-months of age [86]. Typically robust cognitive deficits are not observed in these mice until 6 months or older [108]. These deficits were observed prior to the onset of Aβ deposition (which begins around 6-months of age in these mice), which was not different between APP23 and APP^+^*-ob/ob* mice, strongly suggesting that the cognitive impairments were independent of amyloid burden in the brain. Twelve month-old APP^+^*-ob/ob* on standard chow diet (4% kcal fat) showed decreased brain weights compared to APP^+^, *ob/ob*, and wildtype mice, which suggests that neuronal degeneration may have occurred in these mice; however, actual measures of cell death were not performed [86].

A novel mouse model of vascular/mixed dementia was created by crossing an AD mouse model (APP_ΔNL/ΔNL_ /PS1_P264L/P246L_ knock-in) with a morbidly obese, diabetic mouse model (*db/db*) [109]. These mice displayed significant vascular pathology in the brain, strokes and aneurysms, and learning impairments in the MWM compared to both non-AD diabetic mice and non-diabetic AD mice. Interestingly, no changes were observed in cortical amyloid-beta deposition, or in expression of enzymes (such as insulin degrading enzyme (IDE) and neprilysin) that degrade and clear amyloid-beta, but presenilin expression was increased overall compared to non-diabetic AD mice. In light of these findings, the authors proposed that the cognitive deficits observed were due to vasculature damage and weakening, and subsequent strokes.

Nine-month old female Tg2576 mice on a long-term (5-months) HFD (60% kcal fat) exhibit spatial learning deficits in the MWM, compared to female Tg2576 littermates maintained on a standard low-fat diet (LFD) (10% fat, 70% carbohydrates, 20% protein); specifically, HFD-fed mice show poor *acquisition* [110]. General motor activity was assessed for 24 h one week *prior* to maze learning (no differences were found); however, swim speed *during* the maze testing was not reported, despite the fact that diet-induced obesity can cause hypoactivity in animal models [111]. Additionally, there is evidence that HFD-induced insulin resistance may promote amyloidogenesis in these mice; female Tg2576 mice in the long-term HFD group had increased levels of soluble Aβ_1-40_ and Aβ_1-42_ in the hippocampus, higher Aβ plaque burden in the cortex, increased γ-secretase activity, as well as decreased IDE activity compared to LFD-fed Tg2576 mice [110].

A very high percentage of fat in the diet may be critical for inducing cognitive deficits. Eight- to nine-month old female APP_SWE_/PS1_ΔE9_ mice maintained on a 45% fat diet for 6-months gained significant body weight without disruption of insulin signaling in the brain, changes in Aβ/APP processing, exploratory differences in the T-maze, or spatial memory in the MWM [112]. Twelve-month old, male C57BL/6J mice on a 41% fat diet [92] were similarly not impaired in learning and memory using the Stone T-maze despite significant elevations in body weight and astrocyte reactivity. These mice also showed no elevated microglial reactivity or cytokine levels (TNF-α, IL-6). In contrast, this same study did find impaired cognition and elevated cytokine levels and microglial reactivity in male mice using a higher, 60% fat diet [92].

### Limitations of the Current Studies

The *animal* studies described above [71,86,105,109,110] investigated short- (2 months) or long-term (5 months) HFD-feeding effects in a variety of AD mouse models (APP23, APP_swe_/PS1_ΔE9_, Tg2576, 3xTg) and long-term (6-months) HFD-feeding in male C57BL/6 mice [92], but without comparison of both feeding protocols within the same study. Spatial learning was assessed in some of the studies [86,92,110], but little work was been done to investigate potential cognitive deficits in working memory or measures of anxiety, the latter of which could confound some of the learning measures reported. Additionally, the predominant use of the MWM in HFD-fed mice with significantly greater body weights and fat deposition compared to control mice may present problems: fat mice will float more easily and may spend less time swimming and/or swim more slowly in the maze, which can artificially elevate their latencies during acquisition and retention testing. In fact, given the drastic differences in body weight, hypoactivity in HFD-fed mice is also likely in dry land mazes and other behavioral tests [111]. Whenever possible, behavioral tests should be chosen carefully with a focus on choosing tasks in which locomotor activity differences are less likely to confound the interpretation of the results. If activity differences are found, they should be taken into account when analyzing the data and interpreting cognitive performance. It is also worth noting that deficits in verbal abilities and certain types of memory such as verbal declarative memory, which are found in human obese and T2DM patients with insulin resistance, cannot reasonably be measured in animal populations; however, many other types of learning and memory performance can readily be measured in rodents, such as procedural and spatial acquisition/retention, working- and long-term memory, visual and olfactory memory, conditioned and operant learning, attention, and executive functioning, among others.

## 6. Inflammation Is Related to Cognitive Deficits

HFD-fed, obese mice show increased numbers of CNS astrocytes and microglia, increased levels of CNS macrophage infiltration and activation, as well as a 30% higher ratio of activated *vs.* resting macrophages in the CNS (particularly in the hippocampus) [113]. Additionally, obese mice (both diet-induced and genetic) show regional reactive astrogliosis (as measured by GFAP—glial fibrillary acidic protein) associated with microvessels in the hypothalamus [114]. Twelve-month old male C57BL/6J mice maintained on a very HFD (60% kcal fat, from lard) show learning and memory deficits [92]. These mice also show elevated levels of pro-inflammatory cytokines (TNF-α, IL-6, Monocyte chemotactic protein 1 (MCP-1)), markers of astrocyte reactivity (GFAP) and microglial reactivity (IBA-1) in the cortex, as well as reduced levels of BDNF. In contrast, mice maintained on a 41% fat diet only showed increased astrocyte reactivity (in addition to increased body weight), while demonstrating normal cytokine levels, microglia, *and preserved spatial learning and memory*. This suggests that the cognitive deficits associated with very HFD (60% fat) may be linked to brain inflammation, and specifically, to elevated pro-inflammatory cytokine levels and microglial reactivity.

Learning and memory deficits have also been observed in the rat models of chronic neuroinflammation [115]. In mice, CNS-injected LPS (lipopolysaccharide) increases expression of certain genes important for learning and memory [116]. Additionally, recent work has shown that TNF-α inhibitors can reduce Aβ-plaques and phosphorylation of tau protein in APP/PS1 mice [117] and 3xTg mice [118], as well as ameliorate cognitive deficits associated with chronic inflammation [119].

Several epidemiological, genetic, and experimental studies have found correlations between the severity of chronic inflammation and cognitive impairments in AD [60,120,121]. Levels of IL-6, a pro-inflammatory cytokine, were elevated in post-mortem tissue from diabetic AD brains compared to non-diabetic AD brains [122]. Another post-mortem brain study [123] found that certain inflammatory markers (membrane attack complex (C5b-9) and microglia immune activation (major histocompatability complex class II, MHCII)) were highly correlated with levels of synapse loss in human AD patients, more so than either Aβ-plaque deposition or neurofibrillary tangle formation. Additionally, the brains of high-pathology control patients (those who showed significant Aβ deposition and neurofibrillary tangle formation, but did not show any cognitive deficits prior to death) showed little to no inflammation.

### 6.1. Anti-Inflammatory and Pro-Cognitive Effects of Diet Reversal

While there is a substantial body of research showing that a HFD can lead to obesity, glucose intolerance, IR, increased oxidative stress, inflammation, and cognitive deficits, very limited work has been done to investigate whether, once established, these deficits can be reversed or ameliorated in wildtype mice by switching them from a HFD to LFD. Even less research has been done to address this question specifically in Alzheimer’s mouse models.

Twelve weeks of HFD (60% kcal fat) in 5-week old, skeletally immature C57/BL6J mice produced deteriorations in cancellous bone structure, compressive biomechanical properties, and decreases in trabecular bone volume fraction in the femoral metaphysis. Once mice were switched to a LFD (10% kcal fat) for 12 weeks, the effects on the vertebrae improved to LFD-only levels, but the effects in the femoral metaphysis did not improve [124]. In another study [125], C57/BL6J mice spent 12 weeks on HFD (55% kcal fat), followed by 3 weeks of LFD (14% kcal fat). LFD intervention resulted in a modest weight reduction, improved glucose homeostasis, decreased local inflammation (as measured by TNF-α, IL-6, IL-1b) in the liver, heart and skeletal muscle, and improved insulin sensitivity in the liver; however, LFD did not reduce inflammation in adipose tissue. Markers for CNS inflammation were not investigated.

Using male and female double-mutant APP_Swe/Ind_ mice (Swedish and Indiana mutations), Maesako and colleagues [126] investigated the effects, both alone and in-combination, of exercise (running wheel access) and LFD control interventions on metabolism, Aβ deposition, and spatial learning and memory in the MWM. Mice were fed a HFD (60% kcal fat) at 2–3 months of age for either 20-weeks, or for 10-weeks, followed by 10-weeks of LFD (10% kcal fat). The LFD used also differed from the HFD in percentage of carbohydrates (70% in the LFD *versus* 20% in the HFD). Mice in the exercise condition also received extra environmental enrichment (EE), such as a larger cage space (2.4 times bigger than control housing) and several objects (rotated regularly) in addition to the running wheel. Body weights differed between groups; the APP mice on LFD with running wheel access weighed less than APP-HFD mice, and APP-HFD mice weighed more than APP-controls. There was no synergistic effect observed in the combination treatment group (exercise and LFD). Ten-weeks of LFD ameliorated hyperinsulinemia and hypercholesterolemia, while exercise did not. Both interventions improved learning in the MWM and improved glucose tolerance, decreased β-secretase-mediated APP cleavage as shown by decreased levels of APP *C*-terminus fragments, and reduced soluble Aβ oligomer levels. In a second study by the same authors [127] using male and female APP_Swe/Ind_ mice and a very similar paradigm, 10-weeks of EE was used as an intervention following 10-weeks of HFD (HFD-feeding continued in the presence of EE for 10-weeks). A control group of APP_Swe/Ind_ mice were fed a LFD (10% kcal fat, 70% carbohydrates) for 20-weeks and kept in standard housing. Dietary interventions started at 2–3 months old and the MWM was used to assess spatial learning and memory retention. The EE condition included free access to a running wheel in the home-cage for the entire duration of the study (5-months), in addition to a larger cage space and several other objects (stands, toys, *etc*.) that were rotated on a regular basis. APP-HFD mice stopped gaining weight, and maintained relatively stable body weights after being switched to the EE condition, despite increases in weekly food intake; however, this result is not very surprising given the increased opportunity for physical activity (running wheel access) during the EE intervention. Ten weeks of EE (and exercise) improved HFD-induced fasting serum glucose levels and glucose intolerance in GTTs, but did not improve serum insulin levels during fasting or 60 m after i.p. glucose injection, compared to APP-HFD mice in standard housing. EE/exercise also decreased Aβ deposition in the hippocampus (measured by IHC), reduced levels of soluble and total Aβ_1-40_ (measured by ELISA (enzyme-linked immunosorbent assay)), decreased levels of APP *C*-terminal fragments, and improved learning in the MWM, as measured by decreased escape latencies on day 5 of acquisition.

### 6.2. Limitations of the Previous Studies

Although the beneficial treatment effects found in both studies using AD mice [126,127] are initially exciting, their interpretation is complicated due to the procedure used to assess spatial learning in the MWM. It appears from the acquisition data that very few if any of the mice learned the maze well by the end of acquisition since all groups had average latencies around 35 s and longer from days 1–5. Further, since mice in the 20-week HFD group weighed significantly more than the LFD intervention or exercise mice, multiple measures of locomotor activity should have been reported for all stages of maze testing, as differences could influence measures of learning, such as escape latencies. Time spent floating was not reported, and while swim speed was purported to not differ between groups during the one day of visible platform training, it was not reported at all for the acquisition phase. However, the number of entries to the target quadrant was reported, and APP-HFD mice made significantly fewer entries to the target quadrant, indicating that there may have been locomotor differences during acquisition that could have contributed to the increased latencies. Additionally, meaningful interpretation of memory retention via probe trial performance is not possible if acquisition of the task was not successful in the first place. While both males and females were used in both studies, only the male brains were used and reported for the biochemical analyses, which may have diminished the effects observed since there could be important sex differences. The prevalence of AD is much higher in females than in males [1], and so knowledge of any sex differences in treatment responses is especially important to elucidate. Sex differences have already been reported for the distribution and amount of adipose tissue, with females depositing more subcutaneous fat, and males depositing more gonadal/intra-abdominal fat [128]. Furthermore, female C57BL/6 mice gain less weight, have improved insulin sensitivity, later onset of glucose intolerance, and a reduced inflammatory response (macrophage infiltration and inflammatory gene expression) in subcutaneous and gonadal adipose tissue compared to males during short-term (<20 weeks) HFD-feeding [129,130]; however, some (but not all) of these differences can be diminished or equalized by extending the amount of time spent on HFD and by using diets containing very high percentage of total and saturated fat.

One of the conclusions from the Maesako *et al.* study [126] was that exercise improved memory in the MWM better than diet control. This seems reasonable given that exercise offers some of the same benefits as diet control, such as weight loss and decreased adiposity; however, unlike diet control exercise can also increase neurogenesis and improve cardiovascular health [131], and can induce changes in synaptic and cytoskeletal proteins to different extents in wildtype and AD mutant mice [132]. Furthermore, in rodents, even short-term (6 weeks) environmental enrichment, in the absence of physical activity, has been shown to improve spatial learning and working memory performance, in addition to increasing neurogenesis and synaptogenesis in the dentate gyrus [133]. A potential confound in the procedures, however, is that in the first study [126], mice in the exercise condition also received extra environmental enrichment (larger cage space, variety of additional objects rotated regularly), and in the second study [127], mice in the environmental enrichment condition also received a running wheel. So it is not clear whether exercise or environmental enrichment alone is responsible for the improved learning in either study, or to what degree each treatment contributed to the improvements observed in these groups. Despite the potential confounds in these studies, they are the first evidence that dietary reversal, and/or exercise-induced weight loss can have beneficial effects on cognition in HFD-fed, AD mouse models. These findings need to be confirmed and the mechanisms for cognitive improvements delineated, in order for this work to provide support for potential interventions.

## 7. Discussion

### Limitations of the Current Evidence Linking T2DM with AD

It should be noted that while many of these diseases’ pathological characteristics may overlap, they are in fact two distinct diseases. Amyloid-β plaques and tau neurofibrillary tangles are key pathological hallmarks of AD, which are notably absent in patients with only T2DM. The cognitive deficits seen in non-demented T2DM humans with low risk of dementia (as measured by biomarkers and family history) and animal models of T2DM are much less severe than the progressive cognitive, behavioral and functional impairments seen in AD, especially during the later stages of the disease.

Additionally, the primary causes of each disease are different. Insulin resistance and inflammation induced by obesity are the main causes of T2DM. In contrast, the primary cause of sporadic AD is not definitively known; different theories have been proposed to explain what initially instigates the disease process (*i.e.*, decreased acetylcholine, hyperphosphorylated tau, amyloid-β oxidative stress), but the amyloid hypothesis is the longest standing as well as being the best-studied to-date. This theory posits that AD pathology is primarily driven by increased production and/or decreased clearance of soluble amyloid-beta oligomers (especially the longer chains, Aβ_1-42_ and Aβ_1-40_). These are subsequently deposited and aggregate into plaques contributing to neuronal damage, increasing oxidative stress and mitochondrial damage, triggering inflammatory processes that eventually become sustained and chronic, and lead to increased hyperphosphorylated tau. A relatively new theory that has emerged in the field proposes that vascular dysfunction and oxidative stress in tandem with neuroinflammation occur first and are the primary factors causing AD [134]. This combination of factors then leads to the increased generation of amyloid-β, which in turn, unleashes a cascade of events that exacerbate neuroinflammation, mitochondrial dysfunction and oxidative stress, as well as contributes to the formation of neurofibrillary tangles. HFD-induced obesity and T2DM can promote vascular dysfunction and oxidative stress, in addition to increasing inflammation independently of oxidative stress, so the evidence presented above could also support this theory. Data from several of the studies mentioned above support the proposal that cognitive deficits can derive from non-amyloidogenic changes [86,109].

## 8. Conclusions

Chronic intake of HFDs, obesity, and T2DM are all risk factors for developing dementia, including AD, and individuals with AD are also at increased risk for T2DM. The pathologies of AD and T2DM have many shared characteristics, including chronic inflammation, increased oxidative stress, impaired insulin signaling/IR and other metabolic disturbances, as well as reduced cognitive functioning. AD mouse models fed very HFDs (60% kcal fat), or crossed with genetic mouse models of diabetes (such as *ob/ob* and *db/db*), show early spatial learning and memory impairments. Short-term diet reversal in animal models can improve insulin sensitivity and glucose homeostasis, reduce some markers of peripheral inflammation, and decrease levels of soluble, oligomeric Aβ, and may thereby improve some of the cognitive impairments associated with obesity, IR, and HFDs. More research on shorter- and longer-term diet reversal in humans and animal models is needed to examine whether this lifestyle intervention can effectively reduce brain inflammation and improve neuronal insulin resistance seen in AD and T2DM, and thus ameliorate the cognitive impairments associated with these characteristics. Very little research has been done on the potential benefits of diet reversal/LFD interventions on CNS inflammation and cognitive impairments induced by chronic HFD consumption, which if effective, represents an economical and widely available treatment possibility.

Future studies using a variety of AD mouse models should investigate the effects of diet reversal following long-term HFD consumption on markers of inflammation in brain and peripheral tissues (fat, liver, *etc.*). A wider selection of behavioral tests should be used to evaluate activity levels, cognition, learning and memory, and anxiety-like behavior in both HFD-fed AD mice and those on LFD reversal interventions. While it wouldn’t be expected to reverse AD pathology, LFD interventions may present a real opportunity for improvements in cognitive functioning in obese, preclinical to early stage AD patients since some of the cognitive difficulties initially experienced by this population may actually be caused by obesity’s effects on inflammatory processes and insulin signaling, as opposed to amyloid burden.
